# Multiscale Characterization of Embryonic Long Bone Mineralization in Mice

**DOI:** 10.1002/advs.202002524

**Published:** 2020-09-24

**Authors:** Isabella Silva Barreto, Sophie Le Cann, Saima Ahmed, Vivien Sotiriou, Mikael J. Turunen, Ulf Johansson, Angel Rodriguez‐Fernandez, Tilman A. Grünewald, Marianne Liebi, Niamh C. Nowlan, Hanna Isaksson

**Affiliations:** ^1^ Department of Biomedical Engineering Lund University Lund 22100 Sweden; ^2^ Department of Bioengineering Imperial College London London SW72AZ UK; ^3^ Department of Applied Physics University of Eastern Finland Kuopio 70211 Finland; ^4^ MAX IV Laboratory Lund 22100 Sweden; ^5^ European Synchrotron Radiation Facility Grenoble 38000 France; ^6^ Department of Physics Chalmers University of Technology Gothenburg 41296 Sweden

**Keywords:** bone development, Fourier transform infra‐red microspectroscopy, small‐ and wide‐angle X‐ray scattering, X‐ray fluorescence spectroscopy, X‐ray tomography

## Abstract

Long bone mineralization occurs through endochondral ossification, where a cartilage template mineralizes into bone‐like tissue with a hierarchical organization from the whole bone‐scale down to sub‐nano scale. Whereas this process has been extensively studied at the larger length scales, it remains unexplored at some of the smaller length scales. In this study, the changes in morphology, composition, and structure during embryonic mineralization of murine humeri are investigated using a range of high‐resolution synchrotron‐based imaging techniques at several length scales. With micro‐ and nanometer spatial resolution, the deposition of elements and the shaping of mineral platelets are followed. Rapid mineralization of the humeri occurs over approximately four days, where mineral to matrix ratio and calcium content in the most mineralized zone reach adult values shortly before birth. Interestingly, zinc is consistently found to be localized at the sites of ongoing new mineralization. The mineral platelets in the most recently mineralized regions are thicker, longer, narrower, and less aligned compared to those further into the mineralized region. In summary, this study demonstrates a specific spatial distribution of zinc, with highest concentration where new mineral is being deposited and that the newly formed mineral platelets undergo slight reshaping and reorganization during embryonic development.

## Graphical abstract

Long bones are largely formed through endochondral ossification, where a cartilage template gradually mineralizes into a bone‐like tissue. However, the characteristics of this mineralization process at the smaller length scales remain unclear. Using a range of high‐resolution synchrotron‐based techniques, distribution of molecular and elemental content, as well as reshaping and reorganization of mineral platelets are visualized.

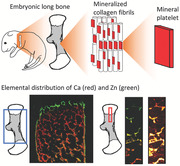

## 1 Introduction

During embryogenesis, the long bones develop, grow, and mineralize into a highly complex and refined design, with the specific goal of being capable of sustaining a range of loading scenarios. This mineralization process results in a hierarchical organization from the whole bone‐scale down to the sub‐nano scale.^[^ ^1^, ^2^, ^3^ ^]^ The resulting mechanical properties of the organ itself are defined by the tissue composition and its structure across all these length scales. However, the characteristics of the mineralization process during the transformation from an unspecialized cartilage template into a complex mineralized design during embryonic long bone development, remains unexplored on many of the hierarchical levels, especially at the smaller length scales.

Long bones are formed through endochondral ossification,^[^ ^4^, ^5^ ^]^ where a cartilage template is gradually mineralized into bone. The mineralization starts in the middle of the rudiment and progresses longitudinally in both directions, mainly governed by the growth plate at both ends. Composed of cartilage matrix and chondrocytes, the growth plate is divided into different zones, all serving a specific purpose.^[^ ^6^, ^7^, ^8^, ^9^ ^]^ Furthest away from the mineralized region is the resting zone, then comes the proliferating zone, hypertrophic zone, and ultimately the mineralization zone. In short, the regions in the growth plates sequentially go through processes such as chondrocyte proliferation, extracellular matrix synthesis, chondrocyte hypertrophy, matrix mineralization, chondrocyte apoptosis, and blood vessel invasion accompanied by osteoblasts and osteoclasts. The speed of longitudinal bone growth is determined by the size and organization of the growth plate, especially by the proliferation rate of chondrocytes and their rate of maturation into hypertrophic cells.^[^ ^6^ ^]^ Simultaneously as the mineralized region grows in length, radial growth of the already mineralized diaphysis and reorganization occurs through intramembranous bone formation and resorption, forming the bone collar.^[^ ^4^, ^5^ ^]^ Intramembranous bone formation occurs through direct mineral deposition by osteoblasts that differentiates directly from mesenchymal stem cells, without a precursor cartilage template. The reader is referred to excellent reviews by Olsen et al. (2000) and Shapiro (2008) for further details.^[^ ^4^, ^5^ ^]^

The morphological and cellular events of endochondral ossification have been studied extensively.^[^ ^4^, ^5^, ^6^, ^8^, ^9^, ^10^, ^11^, ^12^, ^13^ ^]^ Despite this, the pathway of which ions are supplied to and crystallize into hydroxyapatite (HA, Ca5(PO4)3(OH)) at the growth plates is still under debate. Intracellular vesicles with amorphous calcium phosphate (CaP) have been identified in osteoblasts of embryonic mice ^[^ ^10^ ^]^ and chick^[^ ^11^ ^]^ long bones, as well as in hypertrophic chondrocytes.^[^ ^12^ ^]^ The amorphous precursors then crystallize into HA after they have been released into the extracellular matrix (ECM). However, Haimov et al. ^[^ ^13^ ^]^ recently suggested an alternative pathway, through the release of mineral‐containing vesicles directly from the invading blood vessels.

Studies of other sequential events in the ECM during embryonic bone mineralization, however, are still sparse. Recently, an extensive study by Ahmed and Nowlan^[^ ^14^ ^]^ presented crucial changes in the structural organization of collagens during murine embryonic development. Another study recently reported the mineral particles of embryonic mouse long bones to be thin, curved platelets,^[^ ^10^ ^]^ consistent with adult bone where most mineral particles have been found to be one crystal thick, curved, and elongated polycrystalline plates.^[^ ^15^, ^16^ ^]^ Furthermore, Ca has been found to be present in another form than crystalline HA in embryonic mice and the platelet thickness of the bone collar to decrease between embryonic and postnatal mice.^[^ ^17^ ^]^ Also, studies of HA maturation and compositional changes in embryonic rat calvaria have shown increases in mineral to matrix ratios with development.^[^ ^18^, ^19^ ^]^

To assess bone mineralization, a range of high‐resolution tissue characterization techniques exists. X‐ray microcomputed tomography (μCT) is used to determine the bone morphology and structure on the macro/micro scale.^[^ ^20^, ^21^ ^]^ Scattering techniques such as small‐ and wide‐angle X‐ray scattering (SAXS/WAXS) are employed to study the size and structural organization of the mineral platelets at the nanoscale.^[^ ^22^, ^23^, ^24^, ^25^ ^]^ To gain compositional information of bone, Fourier transform infra‐red (FTIR) spectroscopy is a frequently used technique that probes the relative molecular composition and distribution.^[^ ^26^, ^27^, ^28^, ^29^ ^]^ Another spectroscopy‐based technique is X‐ray fluorescence (XRF), which instead quantifies the elemental composition.^[^ ^22^, ^30^ ^]^

All aforementioned techniques can probe structural and compositional characteristics nondestructively, as compared to conventional methods such as, e.g., histology and immunohistochemistry. In addition, they are implemented at synchrotron facilities. Hence, due to the high energy, coherence and flux, these techniques can provide substantially higher resolution, better signal‐to‐noise ratios and shorter acquisition times than conventional lab options.^[^ ^22^ ^]^ Another benefit is that the techniques probe the nanoscale across micrometer sized regions, which both provide a spatial advantage and better account for heterogeneity compared to, e.g., electron microscopy techniques. Furthermore, several of these techniques are often combined at the synchrotron facilities, enabling simultaneous acquisition of different data sets providing complementary information at multiple scales with spatial correlation. Some studies of bone mineralization utilizing combinations of these techniques have emerged: A recent study using synchrotron‐based XRF and diffraction scattering tomography in three dimensions with sub‐micrometer spatial resolution (400 nm) to investigate osteonal biomineralization in humans, found smaller mineral crystals and an increased Ca concentration in the newly formed bone close to the Haversian canal of the osteon.^[^ ^31^ ^]^ Another recent study combining synchrotron‐based X‐ray scattering with Raman spectroscopy, backscattered electron microscopy, histology and μCT to investigate mineralization at the tibial growth plate in young mice and the effect of bone metastasis, found smaller and less organized mineral crystals close to the growth plate than those of the mid‐diaphyseal cortex.^[^ ^32^ ^]^

**Figure 1:**
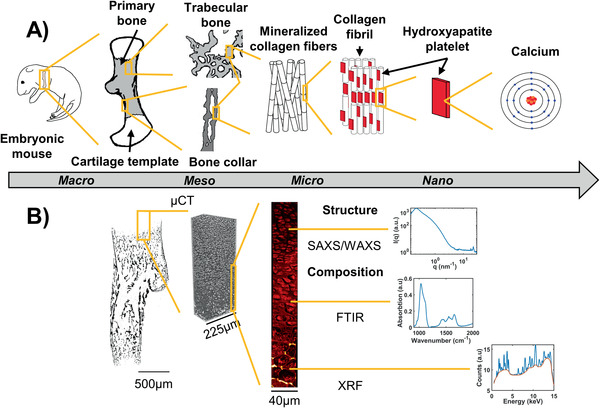
Overview of the multimodal and multiscale approach. A) Scheme of the hierarchical structural organization in embryonic long bone. At the macro‐scale, the rudiment is composed of a cartilage template and a mineralized region originating from the middle of the rudiment (primary bone). At the mesoscale, bone can be divided into two subgroups; trabecular and cortical‐like bone (bone collar). Both subgroups are composed of mineralized collagen fibrils at the microscale. The nanoscaled mineral platelets embedded in the collagen fibril network is mainly constituted by Ca (forming HA: Ca_5_(PO_4_)_3_(OH)). B) Overview of the techniques used in this study to probe the structure and composition of the different length scales. The overall morphology and inner structure were obtained using μCT with 0.65, 0.81 and 1.625 µm spatial resolution. The molecular composition was probed by FTIR with 4.6 µm spatial resolution. The mineral platelet dimensions were determined with a 2 µm spatial resolution using SAXS/WAXS. Elemental composition was evaluated using XRF, at both 2 µm and 200 nm spatial resolutions.

The studies of embryonic bone mineralization utilizing the above mentioned material characterization methods,^[^ ^10^, ^17^, ^18^, ^19^ ^]^ have focused on a limited set of embryonic development stages and in localized regions or bulk measurements, overall limiting the spatial characterization. The present study addresses this knowledge gap, by characterizing the mineralization process during long bone development of mice humeri using a multimodal and multiscale approach. Specifically, we investigated the changes in morphology, composition and structure of mineralizing long bones during embryonic murine development using μCT, FTIR, SAXS, WAXS, and XRF (**Figure** **1**). The approach enabled us to reach high spatial resolution (down to 200 nm) at several different hierarchical length scales across large areas of the humeri. Thus, this study provides unprecedented insight into the hierarchical structural and compositional changes during prenatal ossification.

## 2 Results

### 2.1 Macro‐/Microscopic Characterization of Shape and Inner Structure

**Figure 2:**
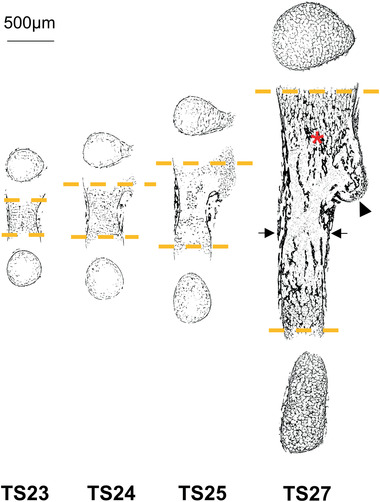
Evolution of morphology and inner structure of embryonic mice humeri. 2D slices of the µCT reconstructions of one representative sample per age group, showing bone mesoscopic distribution of the mineralized (hard) tissue. Separation into cortical‐ and trabecular‐like structures (arrows and asterisk respectively), as well as mineralization of the deltoid tuberosity (arrowhead) can be observed as early as in TS24. The longitudinal slices were acquired at the center of the rudiment and the cross‐sections at the first slices at each end containing bone structure throughout the whole slice (orange dashed lines).

To investigate the changes in rudiment morphology, mice ranging from the start of long bone mineralization (Theiler Stage TS23) to shortly before birth (TS27) ^[^ ^33^ ^]^ were imaged using synchrotron based μCT. A macroscopic transition in shape of the mineralized tissue from a uniform, cylindrical bone cross‐section at TS23, toward an elliptical, proximally to distally twisted shape at TS27 was observed (**Figure** **2**). The beginning of the change into the elliptical shape and slight twisting was noticeable already at TS24. The deltoid tuberosity (Figure 2, arrowhead), to which the deltoid muscle attaches,^[^ ^34^ ^]^ started to mineralize around TS24 and was completely mineralized at TS27. The mineralized regions of the humeri grew in length substantially, with roughly 400% increase between TS23 and TS27, and the separation into a cortical‐like bone collar (Figure 2, arrows) and trabecular‐like inner structures (Figure 2, asterisk) was observed to begin already at TS24.

### 2.2 Molecular Compositional Characterization

**Figure 3:**
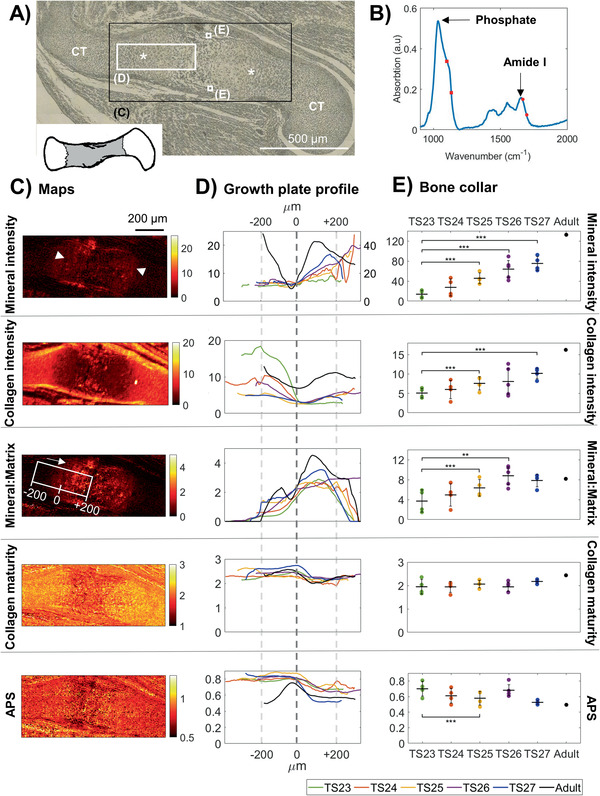
Spatial molecular composition and evolution with age in embryonic mice humeri. A) Optical microscopy image acquired in plain polarized light of a representative section of an embryonic humeri from TS24, where the growth plates are indicated by asterisks (:) and the cartilage template with CT. The black box indicates the total scanned area (C) and white boxes the further analyzed areas, i.e., the growth plate (D) and bone collar (E). B) Representative FTIR spectrum from one scanned point (pixel size 4.6 µm). Arrows point to the phosphate band (corresponding to mineral content) and Amide I band (corresponding to collagen content). The red dots and squares indicate the location of the sub‐bands correlated to collagen maturity and APS, respectively. C) Spatial maps of mineral, collagen, mineral to matrix ratio, collagen maturity and APS from the representative sample in (A). Arrowheads in the mineral map indicate the approximate mineralization fronts of the growth plates. The white box in the mineral:matrix map shows the area evaluated in (D) and the approximate zero‐point. The longitudinal direction is indicated by an arrow. D) The average growth plate profiles per age group (*N* = 4), longitudinally across the approximate area indicated by the white box in the mineral:matrix map (C). Color code corresponds to embryonic age as seen in (E). The zero point (dashed line) represent when mineral:matrix ratio reached 2 or above. Please note that the Adult (black) mineral values are on the secondary axis and that all curves were smoothed after alignment (hence the slightly different mineral:matrix values at 0 µm). E) Average compositions per age group in the bone collar (*N* = 4). Error bars show the 95% confidence interval. Linear mixed effect analysis was used to test for statistically significant differences with age. Statistical significance is indicated with ^::^ *p* = 0.01 and ^:::^ *p* = 0.001.

To evaluate the development of the molecular composition and its distribution, mice at the start of long bone mineralization (TS23) to shortly before birth (TS27), as well as a control adult mouse, were imaged using transmission FTIR microspectroscopy. The proximal and distal growth plates and two regions in the bone collar were analyzed (**Figure** **3**A). From the absorption spectrum (Figure 3B), the mineral content (phosphate peak 1200–900 cm^−1^), collagen content (Amide I peak 1725–1575 cm^−1^),^[^ ^27^ ^]^ mineral to matrix ratio (ratio of phosphate:Amide I), collagen maturity (ratio of the Amide I sub‐bands 1660:1690 cm^−1^) ^[^ ^35^ ^]^ and acid phosphate substitution (APS, ratio of the phosphate sub‐bands 1127:1096 cm^−1^) ^[^ ^29^ ^]^ were calculated to generate the distribution maps (Figure 3C).

An increase in mineral content with development stage was observed both at the mineralization front of the growth plate (Figure 3D, mineral, 0–200 µm) and in the bone collar (Figure 3E, mineral). The collagen content showed a decrease with development stage in the premineralized zone of the growth plate (Figure 3D, Collagen, −200 µm) and remained low at the mineralization front in all developmental stages, but not in the adult (Figure 3D, Collagen, 0–200 µm). In the bone collar however, the collagen content increased with development stage (Figure 3E, Collagen). At the mineralization front of the growth plate, the mineral to matrix ratio showed similar values throughout development (Figure 3D, mineral:matrix, 0–200 µm). In the bone collar on the other hand, the mineral to matrix ratio increased significantly between TS23 and TS26, where it reached a value similar to that of the adult reference (Figure 3E, mineral:matrix). The collagen maturity remained similar throughout development (Figure 3D–E, collagen maturity). No apparent difference in APS with development stage was observed at the growth plate (Figure 3D, APS), but a significant decrease between TS23 and TS25 was found in the bone collar (Figure 3E, APS). By TS27, the APS in the bone collar had reached similar values to that of the adult.

### 2.3 Elemental Compositional Characterization

**Figure 4:**
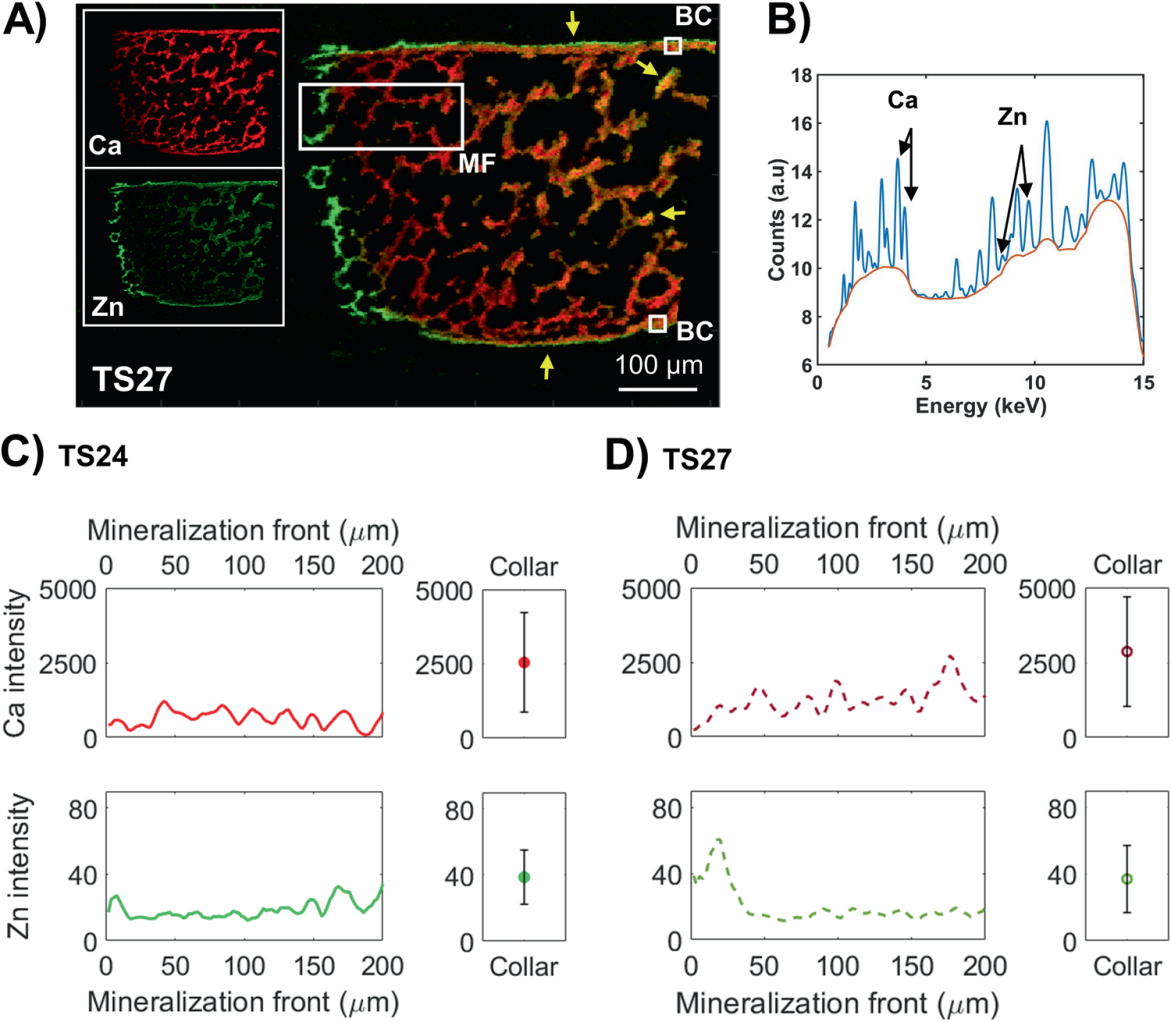
Micrometer‐resolved spatial elemental composition in TS24 and TS27. A) Representative combined Ca and Zn XRF intensity map from TS27 acquired at ESRF, where Ca is red and Zn green (overlap is yellow). Yellow arrows indicate localized regions of high Zn concentrations outlining the bone collar and inner structures. Further analyzed regions of interest are indicated with white boxes; the mineralization front of the growth plate (MF) and the bone collar (BC). B) Representative XRF spectrum from one scanned point (pixel size 2 µm), where the Ca and Zn peaks are indicated with arrows. C) Comparison of average longitudinal profiles of Ca and Zn intensities between the mineralization front of the growth plate and the averages from two areas of 20 × 20 µm^2^ each in the bone collar in TS24 (*N* = 1). Zero point was set to when the Ca intensity reached above 1/10 of the values of the clearly visible mineralized structures. The error bars correspond to the standard deviation of the values within the two areas. D) Comparison of average longitudinal profiles of Ca and Zn intensities between the mineralization front of the growth plate and the bone collar in TS27 (*N* = 1).

To assess the changes in elemental composition and distribution, first the entire mineralized regions of humeri from TS24 and TS27 were evaluated using scanning synchrotron based XRF (**Figure** **4**A). In each point, the elemental concentrations of Ca, P, Zinc (Zn) (Figure 4B) and other trace elements were quantified.

The mineralization front of the growth plate (Figure 4A, white rectangle marked MF) in both samples showed lower Ca content compared to the bone collar (Figure 4A, white box marked BC), which had mineralized a few days earlier (Figure 4C,D, Ca intensity). At the mineralization front of the growth plate in TS27, a slight increase in Ca content was noted toward the more mineralized region (Figure 4D, Ca intensity, 0–200 µm), whereas at TS24 Ca content remained constant throughout the profile (which in this sample also reached the middle of the rudiment) (Figure 4C, Ca intensity, 0–200 µm). In TS27, the Ca amount of the collar was similar to the values reached at ≈200 µm into mineralization (Figure 4D, Ca intensity). The Zn content in both profiles showed a clear local increase just before and at the mineralization front of the growth plate (Figure 4A,C,D, Zn intensity, 0–50 µm), exemplified with the TS27 where the Zn increased with roughly 200% compared to further into the mineralization. Higher local Zn content was also found outlining the edges of the bone collar and inner, trabecular‐like bone structures, most clearly in TS27 (Figure 4A, yellow arrows).

**Figure 5:**
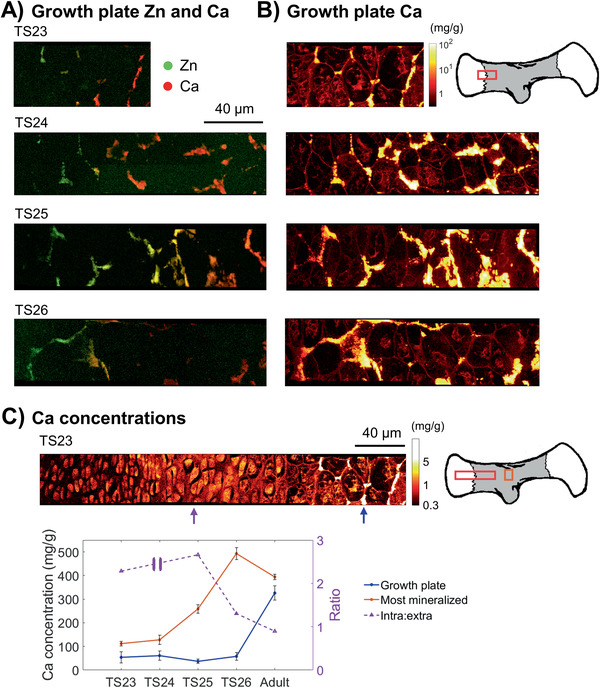
Nanometer‐resolved spatial elemental concentration across growth plates in embryonic mice humeri. A) Combined Ca and Zn XRF intensity map from the mineralization front of the high‐resolution growth plate scans acquired at MAX IV with a pixel size of 200 nm, where Ca is red and Zn green (overlap is yellow). B) Only Ca concentration (logarithmic scale) of the same region of the growth plate, where chondrocytes, ECM and mineralized regions (yellow/white) can be observed. The values are given in mg g^−1^ and the brighter the pixel, the higher the concentration. Note that this scale is set to 0.3–100 mg g^−1^ for all age groups, but that the maximum Ca concentration goes up to around 150 mg g^−1^ for some of them. C) A representative Ca concentration (logarithmic scale) map from the whole growth plate (ROI of growth plate and most mineralized region indicated by the red and orange boxes respectively in the sketch to the right) at TS23. Please note that the scale has been set from 0.3 to 5 mg g^−1^ to better visualize the intracellular concentrations of around 2 mg g^−1^. A comparison of the average Ca concentration of a mineralized cluster at the beginning of mineralization (≈2 chondrocytes into mineralization, blue arrow in growth plate map), the most mineralized regions (bone collar or middle of rudiments, orange box in sketch) and the ratio of the average Ca concentration within and outside three cells of the proliferative zone (purple arrow in growth plate map) with age is shown (*N* = 1). Please note that the ratio is on the secondary *y*‐axis.

To further substantiate the distributions of Ca and Zn within these regions of interest, higher resolution measurements (200 nm step size) of the growth plate and highly mineralized regions were acquired from additional mice from an increased number of development stages (TS23–TS26), as well as from one adult mouse. The intensities from these scans were calibrated to concentrations with a Ca standard. Interestingly, all samples confirmed the localized increase of Zn at the mineralization front of the growth plate consistently across development stages TS23–TS26 (**Figure** **5**A) as observed in the micrometer‐resolved measurements (Figure 4A). The majority of the observed Zn was present in the extracellular matrix in‐between cells and in close association with the newly deposited mineral (Figure 5A,B).

At the initiation of the mineralization front of the growth plate (approximately two chondrocytes, or 40 µm in, Figure 5C, blue arrow) the Ca concentration remained constant across all development stages evaluated (Figure 5C, blue arrow and line; Figure S1, Supporting Information), but very low in comparison to the adult reference. In the most mineralized regions (bone collar) however, the Ca concentration increased between TS23 and TS26, to reach similar levels at TS26 as the cortex of the adult reference (Figure 5C, orange box and line). The ratio between the intra‐ and extracellular Ca concentration averaged over three cells and surrounding ECM in the proliferative zone showed an initially higher intracellular concentration at TS23 and TS25, but approximately the same amount at TS26 as in the adult (Figure 5C, purple arrow and dashed line, secondary axis). Data for the intra‐ to extracellular Ca ratio was excluded for the TS24 sample, since the proliferative zone was not captured by the measurement area.

The distribution of other elements was also investigated, showing that Ca and P overlapped in all mineralized structures and Fe was found in cell‐like patterns in‐between the structures.

### 2.4 Structural Characterization of Mineral Platelets

**Figure 6:**
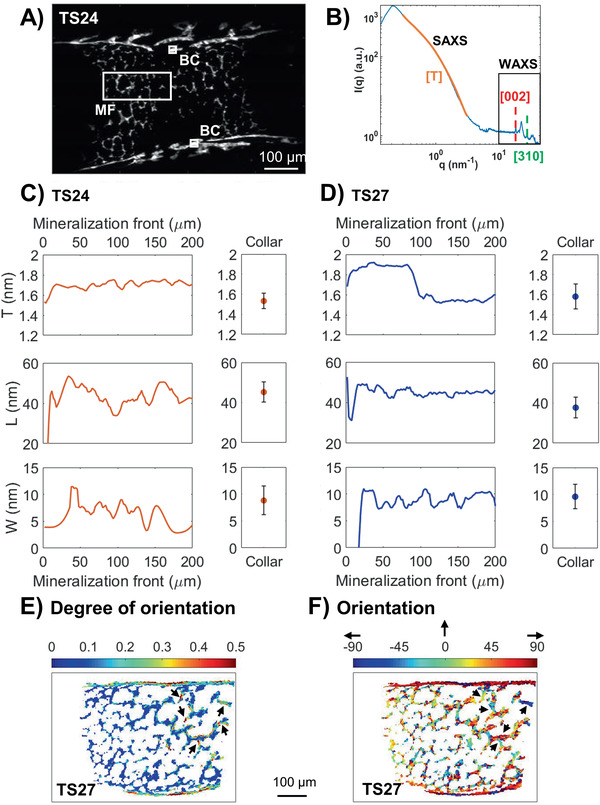
Structural characterization of mineral platelets in TS24 and TS27. A) Symmetric scattering intensity map from TS24 in the *q*‐range of 1.01–1.50 nm^−1^. The white boxes indicate the regions of interest further analyzed for HA dimensions; the mineralization front of the growth plate (MF) and the bone collar (BC). B) Representative scattering curve from one scanned point (pixel size 2 µm), indicating the SAXS regime (*T*‐parameter, orientation) at low *q* and WAXS at higher *q* (*L*‐ and *W*‐parameter). The orange line shows the shoulder which was fitted for the mineral platelet thickness T, the red and green dashed lines shows the peaks fitted for the *L*‐ and *W*‐parameter, respectively. C) Comparison of average longitudinal profiles of mineral platelet dimensions between the mineralization front of the growth plate and the averages from two areas of 20 × 20 µm^2^ each in the bone collar in TS24 (*N* = 1). Zero point was set to when the Ca intensity (acquired by XRF) reached above 1/10 of the values of the mineralized structures. The error bars correspond to the standard deviation of the values within the two areas. D) Comparison of average longitudinal profiles of mineral platelet dimensions between the mineralization front and the bone collar in TS27 (*N* = 1). E) Degree of orientation of the mineral platelets in TS27, where zero is random orientation and one is complete alignment. Arrows point to inner bone structure with similar degrees of orientation as in the collar. F) Actual mineral platelet orientation in degrees in TS27. Arrows point to inner bone structures showing orientations aligned with the major loading axes of the structures. Please note that 0° is defined as up in the vertical direction and ±90° as horizontal (see black arrows).

The presumably ordered and periodic organization of the HA mineral platelets were visualized in the same humeri as the overview XRF measurements (TS24 and TS27), by simultaneously using scanning synchrotron based SAXS and WAXS to evaluate the evolution of platelet size and orientation. The scattering *I*(*q*) curves (**Figure** **6**B) at the mineralization front of the growth plate and in the bone collar (Figure 6A, MF and BC, respectively) provided spatial estimation of platelet thickness (*T*‐parameter) and orientation, as well as length and width of the coherent blocks in the 002‐ and 310‐direction (*L*‐and *W*‐parameter, respectively).

A higher *T*‐parameter was observed at the mineralization front of the growth plate compared to the bone collar (Figure 6C,D,T; Figure S2A, Supporting Information). This difference was observed at both development stages but was especially pronounced in TS27. In TS27, the *L*‐parameter was also higher at the mineralization front of the growth plate as well as in the inner structures compared to the collar (Figure 6D,L; Figure S2B, Supporting Information). Some areas of platelets with lower *L*‐parameters were also found in the bone collar of the TS24 (Figure S2B, Supporting Information) but they were generally of similar values as the newly formed platelets at the mineralization front in both TS24 and TS27 (Figure 6C,D). Platelets with slightly lower *W*‐parameters were observed at the mineralization front compared to the more mineralized regions (Figure 6C,D,W; Figure S2C, Supporting Information).

A higher degree of orientation of the platelets long axis was found in the bone collar at both developmental stages compared to their inner mineralized structures, with TS24 having an overall lower degree of orientation than TS27 (Figure 6E; Figure S3A, Supporting Information). Some parts of the inner mineralized structures in TS27 (Figure 6E, black arrows); however, presented similar degrees of orientation as in the bone collar. A predominant orientation along the major loading axis of the collar was found at both developmental stages (Figure 6F; Figure S3B, Supporting Information), as well as along the axes of some of the inner bone structures in TS27 (Figure 6F, black arrows).

To further evaluate some of these trends, WAXS measurements were also conducted in selected smaller regions of the high‐resolution XRF‐scans (200 nm) of the growth plates and highly mineralized regions from development stages TS23‐TS26. The results confirmed the trends seen in the overview scans described above.

## 3 Discussion

In this study, we assessed long bone mineralization in embryonic mice with a multimodal approach using high‐resolution advanced tissue characterization techniques combined with spatial mapping. The approach enabled us to determine highly resolved spatial distribution over extensive areas not previously reported of the gross morphology, the molecular composition, the mineral platelet dimensions, as well as the basic building blocks of the minerals, Ca and P. Moreover, the approach enabled us to map both mineral platelet dimensions and composition of multiple elements simultaneously at the same spatial positions. Most importantly, it was clearly observed that Zn is distributed in a specific pattern during mineralization, with increased levels just before the immediate mineralization front of the growth plate as well as in other regions where mineral deposition is occurring. Moreover, our results show that the mineralization of long bones is rapid, which is in line with previous data from postnatal development ^[^ ^36^ ^]^ and histological studies of embryonic bone.^[^ ^37^ ^]^ During the four days of development that were studied, the rudiment transformed from an unspecialized shape and inner structure with low Ca content and unordered mineral crystals, into a long bone with a clearly developed morphology, structure and composition almost resembling that of the adult. During this time, the tissue grew extensively in length, organized toward more trabecular‐ and cortical‐like structures and the mineral platelets underwent minor reshaping and started to orient along the major loading axes of these structures.

For the first time to our knowledge, we demonstrate the unique and extensive spatial distribution of Zn in the developing embryonic long bone. This has previously only been reported in postnatal bone development or at the whole bone, organ and cellular levels, where it has been shown to, e.g., stimulate bone formation,^[^ ^38^ ^]^ strength and growth rates,^[^ ^39^ ^]^ as well as affect the morphology of both the whole bones and the growth plates.^[^ ^40^ ^]^ In the current study, Zn was consistently observed at the ossification front of the growth plate at all development stages TS23–TS27 based on data from two separate XRF experiments. Zn was also found to outline the cortices and some inner structures in the micrometer resolved overview scans of TS24 and TS27. While the mechanisms involved is outside the scope of this study, the relation between Zn and mineralization has been extensively studied in postnatal development. Zn is present during bone mineralization as a co‐factor of many matrix metalloproteases (MMPs), such as MMP9, MMP13 and alkaline phosphatase (ALP).^[^ ^41^, ^42^, ^43^ ^]^ MMP13 is specifically expressed by the hypertrophic chondrocytes and osteoblasts proximal to the ossification front, whereas MMP9 is expressed in osteoclasts and highly concentrated at the sites of cartilage resorption in proximity to the ossification front.^[^ ^41^ ^]^ These two MMPs work synergistically for the initiation and development of the primary (growth plate) and secondary ossification sites. ALP is highly present in the mineral containing vesicles at the growth plate and its activity decreases during mineralization, presumably through the loss of Zn at its active site.^[^ ^42^ ^]^ Thus, the clearly local increased Zn concentrations at the growth plate described in this study could be attributed to one or several of these MMPs and an indication of where mineralization occurs. Our finding is also in line with a recent study by Brister et al.,^[^ ^44^ ^]^ which identified particularly high Zn concentrations at sites of ossification in postnatal murine cochlea. Further, increased concentrations of Zn has been found at the tidemark between normal and mineralized cartilage in adult horses ^[^ ^45^ ^]^ and humans,^[^ ^46^ ^]^ presumably as a co‐factor of MMPs.

In addition to its role in many MMPs, Zn can be substituted for Ca in HA.^[^ ^47^ ^]^ A recent in vitro study of human mesenchymal stem cells found that the biomineralization process starts with a Zn‐HA nucleation within the osteoblasts.^[^ ^48^ ^]^ This germinal role of Zn in direct mineralization could explain that Zn outlined the bone collar and inner structures in our study, where intramembranous bone formation is taking place. While we cannot conclusively state what the specific localization of Zn can be attributed to, we can hypothesize that the Zn observed in this study is a combined effect of: 1) MMPs at the growth plate and 2) Zn‐HA nucleation in the bone collar and internal structures. Thus, our study points that further evaluations of the role of Zn during normal development, as well as in pathologies affecting mineralization, are warranted.

Ca was present in low concentrations in close proximity to the hypertrophic chondrocytes of the growth plate, where there was no scattering signal (Figure S4A,B, asterisks, Supporting Information). This finding indicates the presence of Ca in another form than HA, which is in line with similar findings from Lange et al. in embryonic mice femora and tibiae.^[^ ^17^ ^]^ We found for the first time that this was consistent across all the developmental stages investigated. Further, a steeper correlation between scattering and Ca signal in TS24 compared to TS27 was observed (Figure S4C, Supporting Information), indicating a higher presence of amorphous Ca in the younger sample. Recently, Haimov et al. ^[^ ^13^ ^]^ found both membrane‐bound mineral aggregates as well as nonbound amorphous CaP to be deposited in the hypertrophic zone of postnatal murine growth plates from the invading blood vessels. Further, they also observed some osteoblasts in the preossification zone which contained membrane‐bound mineral aggregates. The cartilage then ossified in close proximity to the blood vessel walls as well as the hypertrophic chondrocytes, in a similar pattern as found here (Figure S3A, asterisks, Supporting Information). We also found that there is an increased amount of Ca present within the proliferative cells of the growth plate at the younger stages, which is indirectly in line with the findings by Mahamid et al. ^[^ ^10^ ^]^ and several others which have identified CaP filled vesicles inside the hypertrophic chondrocytes of the growth plate.^[^ ^12^ ^]^ As this study observed Ca both inside the chondrocytes as well as extracellularly, our findings support that the precursor mineral phase is not only extracellularly mediated but might be a combination of both proposed theories.

Rapid increase in the relative mineral to matrix ratio of the bone collar was observed, reaching similar values as the adult bone by TS26. However, both the absolute mineral (phosphate) and collagen (amide I) contents were still far below the amounts found in the adult. This trend indicates an initial rapid deposition of mineral, potentially transitioning into an approximately equal deposition of both mineral and collagen as development continues. As the starting point is an unmineralized cartilage template, the initial rapid mineralization of the rudiment might occur in order to as quickly as possible reach a mechanically viable structure through an optimal compositional ratio. Both mineral and matrix components then increase in content together as the bone continues to reorganize and grow. This plateau in mineral to matrix has also been observed during embryonic development of rat calvaria.^[^ ^19^ ^]^ We also show that the APS of the most recently mineralized regions at the growth plate is higher compared to the more mineralized collar and that this difference remains similar throughout embryonic development. This finding is well in line with previous studies of bone maturation, where higher APS has shown to correlate with areas of new bone formation.^[^ ^49^, ^50^ ^]^

The size of mineral platelets were found to differ between the most recently mineralized regions compared to previously mineralized regions. This was particularly clear at TS27, where the platelets formed in the most recently mineralized regions were thicker, longer and narrower than those of the collar (Figure 4C,D; Figure S2A–C, Supporting Information). At TS24, the spatially less pronounced differences together and constantly lower Ca amount, suggest that formation of new mineral was not just limited to the growth plate. The decreasing trend contradicts the findings in postnatal bone development ^[^ ^25^, ^32^ ^]^ as well as fracture healing,^[^ ^51^ ^]^ where the opposite is seen. However, embryonic development occurs rapidly over a time period of days whereas postnatal development occurs over weeks up to months. Thus, there is a possibility that the mineral platelets reshape differently during prenatal mineralization compared to postnatal. An interesting observation which supports this difference in reshaping, is that the thicker platelets of the newly mineralized regions found in this study, are still thinner than the observed thinnest, less mature mineral platelets at the growth plate of young mice, as found by, e.g., He et al.^[^ ^32^ ^]^ Moreover, our observation is well in line with a study by Lange et al. on the evolution of mineral platelet thickness during development,^[^ ^17^ ^]^ where they found the thickness to decrease in the bone collars between embryonic and postnatal mice. With the much higher spatial resolution and extended areas probed in this study, we show that the platelet thickness also varies regionally within one sample, which indicates that it is not varying depending on the development stage but instead on the local age of the mineralized tissue. The same fitting method for thickness estimation as used in this study was also applied in a recently published study by Törnquist et al.^[^ ^25^ ^]^ conducted on postnatal and young rabbits. By comparing the thicknesses found for the embryonic platelets to their postnatal results, the same decreasing trend between embryonic and postnatal mineral platelet thickness as found by Lange et al.^[^ ^17^ ^]^ in the bone collar can be observed. Moreover, both *L*‐ and *W*‐parameters of the bone collar at the most developed stage found in this study only differ slightly from those of the cortical platelets found in young (10 weeks) rats.^[^ ^24^ ^]^ Thus, the prenatal reshaping of platelet dimensions with tissue age observed in this study all seem to approach the values previously reported in postnatal and young rodents.

Also in line with previously reported postnatal trends,^[^ ^17^, ^25^ ^]^ we identify some localized higher degrees of orientation related to tissue age and possibly also development stage (Figure 4E; Figure S3A, Supporting Information). Despite these localized higher degrees of orientation, most of the embryonic platelets still lacked orientation, which was also indicated in the study by Lange et al.^[^ ^17^ ^]^ Our increased field of view and spatial resolution however, permitted us to identify these localized higher degrees of orientation not captured by their measurements. Moreover, we show for the first time that at TS27 the mineral platelets of the few days older mineralized regions, e.g., bone collar, are aligned along the major loading axes as reported in adult bone.^[^ ^3^, ^25^, ^51^, ^52^ ^]^

Despite the extensive number of data points collected across the bone rudiments (tens of thousands) and the range of complementary high‐resolution techniques used in this study, there is still a major limitation which needs to be addressed. Conducting this extensive mapping at high spatial resolution (2 µm and 200 nm) at synchrotron facilities is highly time consuming and the beamtime available is limited. Thus, we chose to focus on high resolution and larger areas, thereby limiting the number of samples that could be measured for SAXS, WAXS, and XRF. Therefore, we have focused on comparing newly mineralized with more mineralized regions within the same sample, and only report trends across samples, rather than quantitatively comparing groups. By combining data from multiple beamtimes on different samples, we present complementary information with different spatial resolutions over several development stages. Another limitation of this study is that some measurements were performed on thin sections. In FTIR, variation in section thickness would directly affect the spectra and thereby the area of individual peaks (e.g., mineral and collagen content). Therefore, our primary output of the FTIR measurements is ratio parameter (e.g., mineral to matrix). However, as the sections were cut in random order, we expect any thickness effect on mineral or collagen content to be distributed randomly,^[^ ^53^ ^]^ as indicated by the similar distribution of variance observed at all development stages. Despite those limitations, this study has provided new and complementary insight as to how the mineralization of developing long bones proceeds. The clear trends and changes demonstrated here for de novo mineralization during normal development open the possibility of further investigations into abnormal development, e.g., how abnormal muscle loading or nutritional status influences the extent and localization of new bone formation, reshaping and organization.

## 4 Conclusions

A range of high‐resolution imaging techniques were used to probe the evolution of morphology, composition, and structure in developing murine long bones. Rapid mineralization of the humeri occurred over the studied time period of approximately four days. The mineral platelets of the most recently mineralized regions were found to be thicker, longer, narrower and less aligned than the previously mineralized regions and the bone collar, indicating that they undergo slight reshaping and reorganization during development. Moreover, Zn was found to be specifically localized to the growth zones, indicating sites of new mineralization.

## 5 Experimental Section

##### Samples

Thirty‐nine forelimbs from embryonic mice (C57BL/6 strain) were harvested at time points ranging from the first sign of mineralization (approximately embryonic day E14.5) to shortly before birth (approximately E18.5). The mice were categorized into developmental stages based on the Theiler stages morphological criteria,^[^ ^33^ ^]^ ranging from TS23 (approximately E14.5) to TS27 (approximately E18.5). The embryonic forelimbs were carefully dissected and divided into subgroups so all humeri could be assessed with μCT, FTIR, SAXS, WAXS, and XRF respectively (**Table** **1**). Moreover, a female adult mouse hind limb (C57BL/6 strain) was harvested at the age of roughly 3.5 months and used as a mature‐bone control for FTIR and XRF. The experimental protocol was conducted in accordance with European legislation (Directive 2010/63/EU), project license number P39D18B9C.

**Table 1:** The number of humeri evaluated per age group with the different techniques. The techniques used in this study for assessing the embryonic forelimbs were: μCT; FTIR microscpectroscopy; SAXS/WAXS; μXRF/nXRF. Note that the total number of humeri listed here exceeds the total number of mice used, as some humeri were evaluated with several techniques

	TS23	TS24	TS25	TS26	TS27	Adult
μCT	*N* = 4	*N* = 6	*N* = 3		*N* = 4	
FTIR	*N* = 4	*N* = 4	*N* = 4	*N* = 5	*N* = 4	*N* = 1
SAXS/WAXS		*N* = 1			*N* = 1	
μXRF		*N* = 1			*N* = 1	
nXRF	*N* = 1	*N* = 1	*N* = 1	*N* = 1		*N* = 1

When evaluating the macro‐/mesoscale, a scanned field of view covering the whole rudiment was analyzed (Figure 2). When evaluating the composition and structure at the micro‐ and nanoscale, at least one growth plate and two regions in the bone collar were extracted from a larger field of view of the mineralized region and further analyzed (approximate regions visualized by A in Figures 3, 4, 5, respectively). In general, longitudinal profiles going from the start of mineralization (growth plate) to ≈200 µm into the mineralized zone and two roughly 20 × 20 µm^2^ regions in the bone collar were analyzed. In the profile analyses, the zero‐points were defined according to method‐specific threshold values corresponding to the first appearance of mineralized clusters.

##### Microcomputed Tomography (μCT)

Sixteen embryonic forelimbs (Table 1) were placed in plastic tubes filled with ethanol and imaged with μCT. Measurements were conducted at I13‐2 at the Diamond Light Source (Oxfordshire, United Kingdom). Within a larger study, fifteen forelimb samples from TS23, TS24, TS25, and TS27 were scanned, with effective voxel sizes of 0.81 × 0.81 × 0.81 µm^3^ (TS23–TS25) and 1.625 × 1.625 × 1.625 µm^3^ (TS27).^61^ See Table 1 for the number of samples per age group. The shape and structure of the whole humeri and cross‐sections at each end were evaluated. Detailed experimental parameters are available in the Supporting Information. All reconstructions of the projections were done on site using in‐house python scripts.

##### Fourier Transform Infra‐Red (FTIR) Microspectroscopy

Twenty‐one embryonic forelimbs (Table 1) and the adult hind limb (condyle and mid‐diaphysis) were embedded in paraffin, longitudinally sectioned at 3 µm thickness using a microtome (Leica Microsystems RM2255, Germany) and placed on barium fluoride (BaF_2_) windows. The paraffin was dissolved using xylene and the forelimbs were scanned with FTIR microspectroscopy using a Bruker tensor 27 spectrometer coupled to a Bruker Hyperion 3000 IR microscope (Bruker Corp, USA) with a 64 × 64 focal plane array detector (FPA). The acquired spectra per scanning point were binned 2 × 2, resulting in a final pixel size of 4.6 × 4.6 µm^2^. Detailed experimental parameters are available in the Supporting Information.

Mineral and collagen contents were determined from the linearly baseline corrected phosphate (1200–900 cm^−1^) and Amide I (1725–1575 cm^−1^) bands, respectively ^[^ ^27^ ^]^ (Figure 3B). The mineral to matrix ratio was calculated as the ratio between the mineral and collagen content. Collagen maturity, which is related to the ratio of nonreducible/reducible collagen crosslinks, was determined from the ratio of the sub‐bands 1660/1690 cm^−1^.^[^ ^35^ ^]^ APS, which has been demonstrated to be higher in developing tissues, was computed as the ratio of the sub‐bands 1127/1096 cm^−1^.^[^ ^29^ ^]^ Growth plate profiles were acquired by averaging over 147.2 µm in the transversal direction per sample, then aligning all profiles to the mineral to matrix ratio of 2 (zero‐point) and ultimately average all per age group. When there were two growth plates present in one sample, both were measured and averaged per sample before averaging with the rest of the age group. The bone collar compositions were averaged over two areas of 23 × 23 µm^2^ each per sample. All preprocessing and spectral analyses were conducted using custom‐written scripts in MATLAB (R2018b, The Mathworks Inc., US).^[^ ^54^, ^55^ ^]^

##### X‐Ray Fluorescence (XRF) Microscopy

Two embryonic forelimbs, from development stages TS24 and TS27 (Table 1), were embedded in polymethylmethacrylate (PMMA), longitudinally sectioned at 3 µm thickness using a microtome (Leica EM UC7 ultratome, Leica Microsystems, Germany). The sections were fixed at each corner to silicon nitride (Si_3_N_4_) windows using transparent nail polish. Scanning XRF measurements were conducted at the hard X‐ray microfocus beamline ID13 of the European Synchrotron Radiation Facility (ESRF, Grenoble, France) with a step size of 2 × 2 µm^2^. Detailed experimental parameters are available in the Supporting Information.

The XRF spectrum was collected for each scanned point (Figure 4B) using a single element Vortex EM detector (Hitachi High‐Technologies Corp, US), at a sample‐detector distance of 20 mm and at an angle of ≈70° with respect to the incident X‐ray beam.

Additionally, higher resolution XRF measurements of the growth plates and most mineralized regions of four other forelimbs (Table 1) and an adult hind limb were also conducted at the hard X‐ray nanoprobe beamline NanoMAX at the 3 GeV storage ring of MAX IV Laboratory (Lund, Sweden),^[^ ^56^, ^57^ ^]^ using a beam energy of 14 keV (*λ* = 0.8856 Å), exposure time of 0.12 s and a step size of 200 × 200 nm^2^. The XRF spectrum was collected for each scanned point using a XR‐100SDD detector (Amptek Inc., US) at a sample‐detector distance of 12 mm and at an angle of ≈78° with respect to the incident X‐ray beam. For these measurements, samples were embedded in paraffin, longitudinally sectioned at 3 µm thickness using a microtome (Leica Microsystems RM22255, Germany), and placed on Si_3_N_4_‐windows, which were fixed on brass holders using carbon tape. Furthermore, in contrast to the measurements conducted at ESRF, a piece of NIST SRM 613 (1 mm thickness) (NIST, US) was also scanned and used as Ca reference to calibrate the concentrations.

From both XRF experiments described above, the spatial distribution of elemental intensities were calculated using the XRF analysis program PyMCA (5.4.2) ^[^ ^58^ ^]^ (linear SNIP background subtraction, stripping, scattering, escape peaks and pile‐up). The intensity distribution of Ca, the major component of HA, was further investigated, as well as P and common trace elements such as Zn and Fe.^[^ ^47^ ^]^ The XRF spectra contained signals from more elements but no specific patterns or correlations with Ca were found for these and thus they were not further evaluated. For the higher resolution scans (200 nm step size, MAX IV experiment), concentration calculations were first calibrated in PyMCA for Ca by the spectra acquired from a standard with known Ca amount. To investigate if Ca was only present in the ECM of the growth plate or could be originating from within the cells, the ratio between average intracellular Ca concentration within proliferative cells and the average concentration of the surrounding ECM was estimated from the high‐resolution scans of the growth plates. As the amount of clearly identifiable proliferative cells captured by the scans across the growth plates were limited, three cells per stage were chosen (except for TS24, where there were none present in the scan). Analyses of the element spatial distribution maps generated by PyMCA were conducted using custom‐written scripts in MATLAB (R2018b, The Mathworks Inc., US).

##### Scanning Small‐ and Wide‐Angle X‐Ray Scattering (SAXS and WAXS)

SAXS and WAXS measurements were conducted simultaneously as the XRF measurements at ESRF, with the same step size of 2 × 2 µm^2^ and the same ROIs. WAXS measurements were also acquired in smaller regions of the high‐resolution measurements at MAX IV with 5 s exposure time and with the same step size of 200 × 200 nm^2^. These scattering patterns were collected with a Pilatus 1M detector (Dectris, Switzerland) at a sample‐detector distance of 384 mm.

From the acquired scattering patterns, the beam stop was masked away and the 2D scattering pattern was azimuthally integrated to generate 1D scattering curves *I*(*q*) per scanning point. From the WAXS regime of the acquired *I*(*q*) scattering curves (*q*‐range of ≈10–30 nm^−1^), the length of coherent blocks in the 002‐direction (*L*‐parameter) and width in the 310‐direction (*W*‐parameter) of the mineral platelets were determined. The *L*‐parameter was extracted from the full width at half maximum (FWHM) of the 002‐reflection, positioned at *q* = 1.78–1.88 nm^−1^ and the *W*‐parameter was obtained from the FWHM of the 310‐reflection, positioned at *q* = 2.6–2.9 nm^−1^, as described by Turunen et al.^[^ ^24^ ^]^ (Figure 4B) and detailed in the Supporting Information. The predominant orientation, degree of orientation and mineral platelet thickness (*T*‐parameter) were determined from the SAXS regime of the scattering curves (*q*‐range of ≈0.1–10 nm^−1^). The predominant orientation was obtained from 16 azimuthally integrated angular increments in the *q*‐region 1.01–1.5 nm^−1^ and the degree of orientation was calculated as the ratio between the anisotropic scattering and the total scattering within the same region.^[^ ^59^ ^]^ The *T*‐parameter was obtained through weighed iterative curve fitting of the *I*(*q*) scattering curve in the *q*‐region 0.3–3.5 nm^−1^, as described by Turunen et al.,^[^ ^51^ ^]^ according to the model developed by Bünger et al.,^[^ ^60^ ^]^ detailed in the Supporting Information. This model is based on the assumption that the scattering is dominated by the mineral and that it has the shape of a platelet, where two dimensions (length and width) are considered infinite in relation to the probed third dimension (thickness). All WAXS and SAXS analyses were conducted using custom‐written scripts in MATLAB (R2018b, The Mathworks Inc., US).^[^ ^24^, ^51^, ^59^ ^]^

##### Statistics

The SAXS, WAXS, and XRF data were evaluated qualitatively due to the small number of samples per group. Regarding the FTIR data, linear mixed effects analysis was used to test for statistically significant differences with development stage, while taking into account the high number of measurement points per sample (R Studio, v1.2.1335, RStudio, Inc.). The compositional variable was the response/dependent variable, age group was considered a fixed effect, and sample was defined as random effect. Assumptions of homoscedasticity and normality were considered to be met after visual inspection of the residuals. The model was compared against a null model, where the fixed effect was omitted, using a likelihood ratio test in order to test the significance of changes with development stage.

## Conflict of Interest

The authors declare no conflict of interest.

## Author Contributions

I.S.B., S.L.C., N.C.N., and H.I. contributed in study design; I.S.B., S.L.C., V.S., N.C.N., and H.I. contributed in study conduct; I.S.B, S.L.C., S.A., U.F., A.R.F., T.G., M.L., N.C.N., and H.I. contributed in data collection; I.S.B., S.L.C., T.G., A.R.F., and M.J.T. contributed in data analysis; I.S.B., S.L.C., M.L., N.C.N., and H.I. contributed in data interpretation; I.S.B. contributed in drafting manuscript. All authors revised the manuscript content and approved the final version of the manuscript. I.S.B. and H.I. take responsibility for the integrity of the data analysis.

## Supporting information

Supporting Information
