# Supplementary material for: Multiscale Characterization of Embryonic Long Bone Mineralization in Mice
Source: Adv Sci (Weinh). 2020 Sep 24;7(21):2002524. doi: 10.1002/advs.202002524 (PMC7610310; doi:10.1002/advs.202002524)
Supplement: Supplementary file 1 — Supporting Information [file ADVS-7-2002524-s001.pdf]

© 2020 Wiley-VCH GmbH

ADVANCED SCIENCE
Open Access

Supporting Information

for *Adv. Sci.,* DOI: 10.1002/advs.202002545

Multi-scale characterization of embryonic long bone mineralization in mice

Isabella Silva Barreto, Sophie Le Cann, Saima Ahmed, Vivien Sotiriou, Mikael J. Turunen, Ulf Johansson, Angel Rodriguez Fernandez, Tilman A. Grünewald, Marianne Liebi, Niamh C. Nowlan, Hanna Isaksson

Multi-scale characterization of embryonic long bone mineralization in mice

Isabella Silva Barreto, Sophie Le Cann, Saima Ahmed, Vivien Sotiriou, Mikael J. Turunen, Ulf Johansson, Angel Rodriguez Fernandez, Tilman A. Grünewald, Marianne Liebi, Niamh C. Nowlan, Hanna Isaksson

Supplemental Data – Methods

μCT - experimental details

At I13-2, Diamond Light Source, the μCT measurements were conducted using a beam energy of 25 keV with 70 ms exposure time, at a sample-detector distance of 190 mm and 1900 projections were acquired over 180° (with angular increments of 0.015°). The projections were recorded using a PCO.edge 5.5 camera with objective lenses 8x and 4x for the two age groups respectively, resulting in effective voxel sizes of 0.81 μm for TS23-TS25 and 1.625 μm for TS27. Full experimental description is available by Pierantoni et al. in [1].

FTIR Microspectroscopy - experimental details

FTIR measurements were conducted in transmission mode, using a Bruker tensor 27 spectrometer coupled to a Bruker Hyperion 3000 IR microscope (Bruker Corp, USA) with a 64x64 focal plane array detector (FPA). Data was acquired using a spatial resolution of 2.3 μm, spectral resolution of 4 cm^{-1} and with 64 repeated scans. IR spectra with a bandwidth of 2000 cm^{-1} to 900 cm^{-1} were recorded. Spectra were acquired to cover the entire mineralized region when possible, together with a reference spectrum on an empty window from all samples. All spectra were baseline corrected and the background spectra was subtracted (OPUS, version 7.2, Bruker Corp, USA).

Micrometre resolved SAXS, WAXS and XRF synchrotron measurements (ESRF) - experimental details

SAXS, WAXS and μXRF measurements were conducted through raster scanning with a beam energy of 13 keV (λ = 0.9537 Å, q-range 0.11-45 nm^{-1}), step size of 2 μm and exposure time of 50 ms. At each scanning point, the scattering pattern (SAXS and WAXS) was collected with an Eiger 4M detector (Dectris, Switzerland) at a sample-detector distance of 93 mm. The fluorescence spectra were collected for each scan position using a single element Vortex EM detector (Hitachi Inc., US) at a sample-detector distance of 25 mm and at an angle of approximately 70° with respect to the incident x-ray beam.

Calculation of HA dimensions

The beam stop was masked away and the 2D scattering pattern was azimuthally integrated to generate 1D scattering curve I(q) per scanning point over 360° azimuthal angle when calculating the mineral platelet dimensions and 16 angular increments when calculating pre-dominant orientation and degree of orientation.

To calculate the length and width of coherent blocks in the [002] and [310] directions, i.e. the L- and W-parameter, both peaks of the integrated scattering curve were fitted using a Gaussian-Lorentzian curve. The FWHMs of the fits were used to calculate the crystal dimensions through Scherrer's equation:

$$D = \frac{K\lambda}{\beta_{1/2}\cos(\theta)} \quad (1)$$

Where D is the average size of the dimension related to the corresponding reflection, K is a constant describing the crystal shape (typically 0.9) [2,3], λ the x-ray wavelength, $\beta_{1/2}$ is the FWHM and θ the scattering angle. The peak broadening due to the instrument was considered negligible.

When estimating the mineral platelet thickness, the model developed by Bünger et al.[4] assumes that the platelet has infinite size in two dimensions and a finite size in one dimension (thickness T). The form factor P(q) of this mineral platelet then becomes:

$$P(q) = \frac{1}{q^2}\left(\frac{\sin(qT/2)}{qT/2}\right)^2 \quad (2)$$

The HA mineral crystals most likely vary in thickness and polydispersity was thus included, assumed to follow a Schulz-Zimmer distribution. Then the average form factor $P_{av}(q)$ instead becomes:

$$P_{av}(q) = \frac{\int_0^\infty T^2 P(q) D(T,T_{av}) dT}{\int_0^\infty T^2 D(T,T_{av}) dT} \quad (3)$$

where D(T, T_{av}) is the Schulz-Zimmer distribution of the thickness. Moreover, the mineral platelet cannot occupy the same place as another and thus the excluded volume effect also needs to be taken into account. This was taken into account through inclusion of the random phase approximation (RPA) in the calculations of the effective form factor $P_{eff}(q)$:

$$P_{eff}(q) = \frac{P_{av}(q)}{1+vP_{av}(q)} \quad (4)$$

where v is a adjustable parameter, increased with volume fraction. At longer length scales, i.e. at low q, there are inhomogeneities in the electron density due to e.g. closely positioned mineral crystals which results in increased intensities not described by the model so far. To be able to fit the curve properly at lower q, an effective fractal structure factor $S_{frac}(q)$was introduced:

$$S_{frac}(q) = 1 + A_{frac}q^{-\alpha} \quad (5)$$

where α is the fractal dimension of the fluctuations. A background term B was also added to the weighed fit and the initial guess was evaluated from the Porod-region where $I(q) \sim Pq^{-4}$ according to Porod's law. Thus, the final expression for the total scattering intensity used for evaluating the platelet thickness (in the q-region 0.3-2.5 nm^{-1}) was:

$$I(q) = P_{eff}(q)S_{frac}(q) + B \quad (6)$$

References

1. Pierantoni M, Le Cann S, Sotiriou V, Bodey AJ, Nowlan NC, Isaksson H. How muscle contractions shape embryonic bones: A phase-contrast enhanced synchrotron X-ray tomography study [abstract]. In: Orthopaedic Research Society 2020 Annual meeting. Phoenix (AZ); p. Abstract Nr 0219.

2. Acerbo AS, Kwaczala AT, Yang L, Judex S, Miller LM. Alterations in Collagen and Mineral Nanostructure Observed in Osteoporosis and Pharmaceutical Treatments Using Simultaneous Small- and Wide-Angle X-ray Scattering. Calcif Tissue Int. 2014;95:446–56.

3. Lange C, Li C, Manjubala I, Wagermaier W, Kühnisch J, Kolanczyk M, Mundlos S, Knaus P, Fratzl P. Fetal and postnatal mouse bone tissue contains more calcium than is present in hydroxyapatite. J Struct Biol. 2011;176:159–67.

4. Bünger MH, Oxlund H, Hansen TK, Sørensen S, Bibby BM, Jesper •, Thomsen S, Bente •, Langdahl L, Besenbacher F, et al. Strontium and Bone Nanostructure in Normal and Ovariectomized Rats Investigated by Scanning Small-Angle X-Ray Scattering. Calcif Tissue Int. 2010;86:294–306.

Supplemental Data – Figures

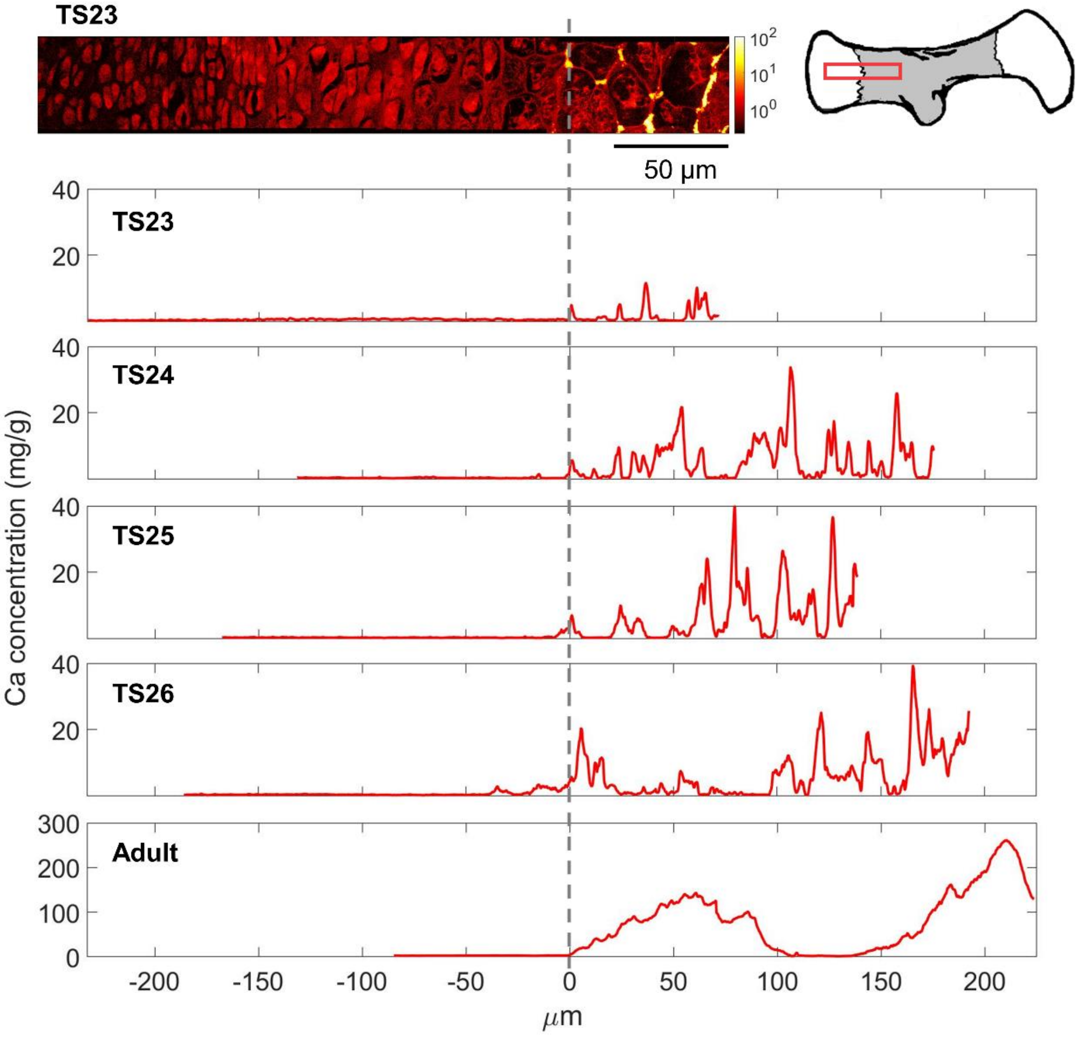

Supplemental Figure S1. Average Ca concentration profiles longitudinally across the growth plates. All profiles are aligned according to when the Ca concentration reached 5mg/g or above (zero-point, dashed line). Please note that the Adult y-axis is substantially different.

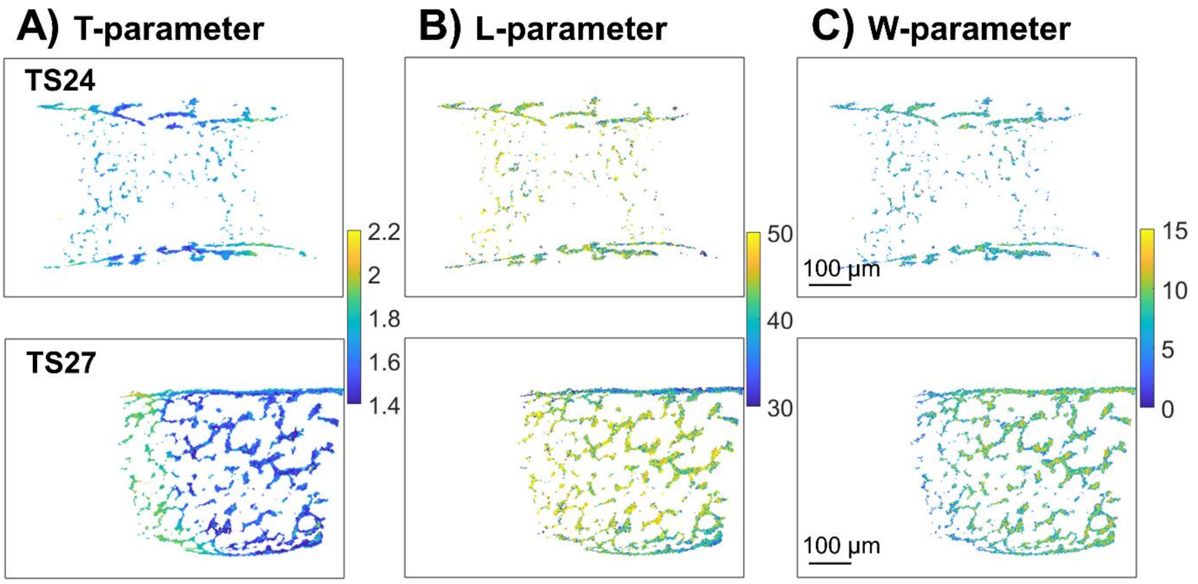

Supplemental Figure S2. Spatial distribution of HA crystal dimensions in TS24 and TS27. Spatial distribution maps of A) T-parameter, B) L-parameter and C) W-parameter. The T-parameter corresponds to the estimated mineral platelet thickness and the L- and W- parameter corresponds to the length and width of coherent blocks in the 002- and 310-direction, respectively. All dimensions are given in nm and the pixel size is 2μm.

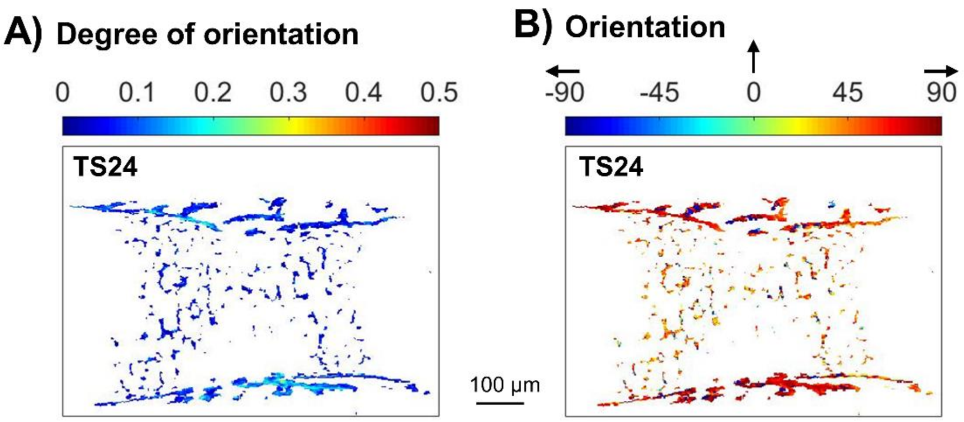

Supplemental Figure S3. Spatial orientation analysis. A) Degree of orientation of the mineral platelets at TS24, where zero is random orientation and one is complete alignment. Pixel size of 2μm. B) Actual mineral platelet orientation in degrees. Please note that 0° is defined as up in the vertical direction and ±90° as horizontal (see black arrows).

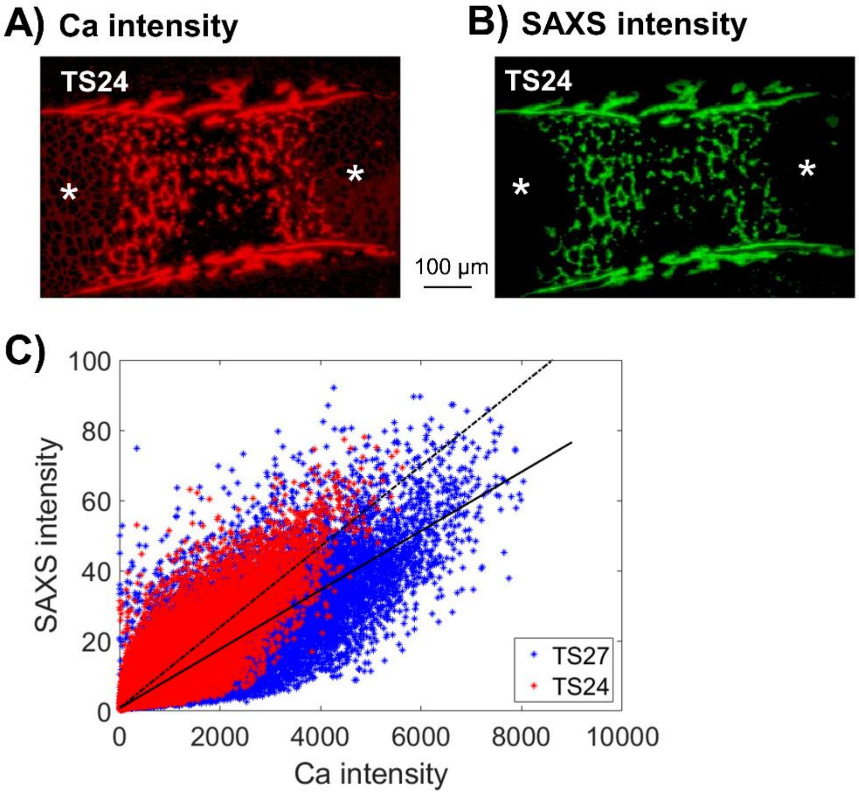

Supplemental Figure S4. Comparison of HA and Ca distributions in embryonic humeri. A) A representative Ca intensity map from TS24. Logarithmic scale normalized to 75% of the maximum value. The brighter the pixel, the higher the intensity. B) Representative symmetric scattering intensity map from TS24, in the q-range of 1.01-1.50nm^{-1}. Logarithmic scale normalized to 75% of the maximum value. Regions where there are Ca signal present but no scattering are indicated in both (A) and (B) by asterisks. C) Symmetric scattering intensity as a function of Ca intensity from both TS24 and TS27.
